# High-Resolution Aerial Imagery Semantic Labeling with Dense Pyramid Network

**DOI:** 10.3390/s18113774

**Published:** 2018-11-05

**Authors:** Xuran Pan, Lianru Gao, Bing Zhang, Fan Yang, Wenzhi Liao

**Affiliations:** 1 Key Laboratory of Digital Earth Science, Institute of Remote Sensing and Digital Earth, Chinese Academy of Sciences, Beijing 100094, China; 201611901006@stu.hebut.edu.cn (X.P.); zb@radi.ac.cn (B.Z.); 2 School of Electronics and Information Engineering, Hebei University of Technology, Tianjin 300401, China; 201621901026@stu.hebut.edu.cn; 3 Department of Telecommunications and Information Processing, Ghent University, 9000 Ghent, Belgium; wliao@telin.ugent.be

**Keywords:** high-resolution aerial imageries, semantic segmentation, densely connected convolutions, pyramid pooling module

## Abstract

Semantic segmentation of high-resolution aerial images is of great importance in certain fields, but the increasing spatial resolution brings large intra-class variance and small inter-class differences that can lead to classification ambiguities. Based on high-level contextual features, the deep convolutional neural network (DCNN) is an effective method to deal with semantic segmentation of high-resolution aerial imagery. In this work, a novel dense pyramid network (DPN) is proposed for semantic segmentation. The network starts with group convolutions to deal with multi-sensor data in channel wise to extract feature maps of each channel separately; by doing so, more information from each channel can be preserved. This process is followed by the channel shuffle operation to enhance the representation ability of the network. Then, four densely connected convolutional blocks are utilized to both extract and take full advantage of features. The pyramid pooling module combined with two convolutional layers are set to fuse multi-resolution and multi-sensor features through an effective global scenery prior manner, producing the probability graph for each class. Moreover, the median frequency balanced focal loss is proposed to replace the standard cross entropy loss in the training phase to deal with the class imbalance problem. We evaluate the dense pyramid network on the International Society for Photogrammetry and Remote Sensing (ISPRS) Vaihingen and Potsdam 2D semantic labeling dataset, and the results demonstrate that the proposed framework exhibits better performances, compared to the state of the art baseline.

## 1. Introduction

In the past few years, image analysis has benefited from deep convolutional neural networks (DCNN), which have been widely applied in image processing tasks, ranging from image classification to object recognition, image super-resolution and semantic segmentation [1,2,3]. Semantic segmentation (i.e., the assignment of a semantic label to each pixel of an image) of high-resolution remote sensing imagea plays a key role in certain application fields, such as urban and agricultural planning, economic forecasting, and more. However, the increasing spatial resolution of remote sensing images does bring about specific challenges. For instance, higher spatial resolution brings out more details and tiny Earth objects, as well as incurring classification ambiguities.

Over the past few decades, methods based on statistical features [4] have been proposed for image classification and detection, such as the maximum likelihood method and K-means. Some methods based on shallow machine learning [5,6,7] support vector machines and have been employed in this task as well, like the neural network. However, most of these traditional methods have some obvious shortcomings. For instance, they mainly depended on low level features or cannot deal with large amounts of training samples. Some new methods, like sparse representation [8] and object-oriented classification [9], have raised and achieved remarkable performance. However, the lack of high-level features hampers the improvement of classification accuracy.

Based on DCNNs, many semantic labeling networks are proposed to perform remote sensing image classification. The deeper structure of DCNNs have wider vision and impressive features of high-resolution remote sensing imagery, which helps networks handle the large intra-class variance and small inter-class differences caused by the increasing spatial resolution.

Researchers first introduced patch-based DCNNs to make pixel predictions of input images. This was accomplished by transforming a whole-image classification network into a semantic labeling network. Furthermore, researchers have utilized patch-based CNNs to predict the classification maps of high-resolution aerial images [10]. In order to further improve the performance, the prediction results of random forest classifiers were combined by using a conditional random field. However, the disadvantages of this method, such as the limited receptive areas and huge computational overheads, soon surpassed the pixel-based method. Volpi et al. [11] compared the patch-based networks with the pixel-based network in the semantic labeling of high-resolution aerial images, and indicated that the pixel-based network was superior in both precision and efficiency. Moreover, they proposed a down-sample-then-up-sample architecture with learnable deconvolutions, which provided a remarkable improvement in this task when compared with the state-of-the-art baseline. Liu et al. [12] proposed to train a fully convolutional network (FCN) [13] on color-infrared (CIR) images, whilst light detection and ranging (LiDAR) data were also considered to benefit the recognition of the Earth object with height information. The inference results of the multi-sensor data were then combined by using higher order conditional random fields (CRFs).

Sherrah et al. [14] also utilized both CIR images and LiDAR data. In their work, a hybrid FCN is trained on two kinds of data, of which FCNs are modified into a no-down-sampling form to preserve output resolution. However, although the no-down-sampling FCN brought a slight improvement in accuracy, it occupied significant video memory usage. Moreover, the limitation of the receptive field makes it outperform the down-sample-then-up-sample architectures.

For high-resolution remote sensing image segmentation, Li et al. [15] proposed DeepUNet. The DeepUNet contained contracting path and expansive path, which followed the architecture of U-Net [16]. In addition, two novel blocks with both U-connection and plus connection were brought to the network to further improve the segmentation performance.

Audebert et al. [17] proposed to train the dual-stream SegNet on multi-sensor data by fusing them together with a residual correction. Additionally, a multi-scale prediction was adopted to introduce different receptive cell sizes into the network. This method achieved significant improvement in the semantic segmentation of remote sensing data when compared with the standard SegNet [18].

Maggiori et al. [19] proposed a CNN architecture with multi-layer perceptron (MLP) to provide fine-grained segmentation maps of high-resolution aerial images. They fused multi-resolution feature maps using MLP to mitigate the recognition/localization trade-off, thus receiving better performance than the skip and unpooling network.

Liu et al. [20] constructed a new architecture, which is called hourglass-shape network, for semantic labeling of high-resolution aerial images. The architecture followed an encoder-decoder paradigm, which introduced inception layers and residual layers to enrich both spatial and contextual information. Nevertheless, the accuracy of the results is still limited due to the simple multi-sensor/resolution feature and fusion strategy.

In short, multi-sensor fusion is a challenging problem in the context of high-resolution aerial image classification. Some methods [11,19,20] stacked multi-sensor data, including CIR images and LiDAR data, as one input vector to train the networks. However, this rough combination of multi-sensor data at the first layer leads to classification ambiguities of certain objects and may also suffer from an information loss problem. Other methods [12,14,17] were proposed to train separable networks for CIR images and LiDAR data, and fused the features at an early or late stage, which ultimately suffered from the multi-fold increase of parameters.

**Figure 1:**
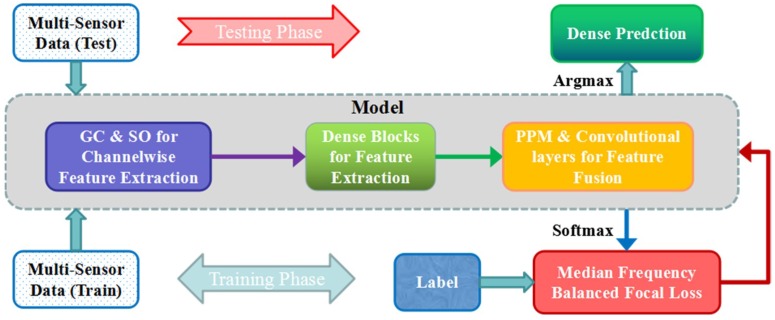
The general illusion of the training and testing procedure for a dense pyramid network (DPN). GC: group convolutions, SO: shuffling operation, PPM: pyramid pooling module.

The existing DCNNs for semantic segmentation of high-resolution remote sensing images suffer from the insufficient spatial and contextual information, as the multi-sensor data fusion is not always better than any single data source. To overcome these problems, we propose a dense pyramid network (DPN) to obtain fine-grained classification maps of high-resolution aerial images. We separately applied group convolutions and shuffling operation to each channel of input to take full advantage of multi-sensor information. Furthermore, densely connected convolutions are introduced to deepen the network, achieving a wider receptive field, and pyramid pooling modules combined with two convolutional layers fuse multi-resolution feature maps at the back end of the network. Additionally, median frequency balanced focal loss (MFFL) is utilized in the training phase to deal with the class imbalance problem, while also forcing the network to attend to the hard-classified samples. The general procedure of the proposed method is shown in Figure 1.

The main findings from of our work can be summarized as follows:

- A DPN is proposed to deal with the semantic segmentation of high-resolution aerial imagery. The architecture based on densely connected convolutions and pyramid pooling modules have wider receptive fields, and can also fuse multi-resolution features through a global contextual prior manner.
- Group convolutions and shuffling operation are set at the beginning of the network to process the multi-sensor data in channel-wise. These two operations can help the network preserve more information from multi-sensor data, while also ensuring that the information flows between channels.
- Median frequency balanced focal loss is applied to deal with class imbalance problem, as well as to reduce the relative loss for well-classified examples and to put more focus on hard, misclassified examples. In particular, we add a constraint on the median frequency weight to mitigate the overreacting problem of certain class.

## 2. Methods

**Figure 2:**
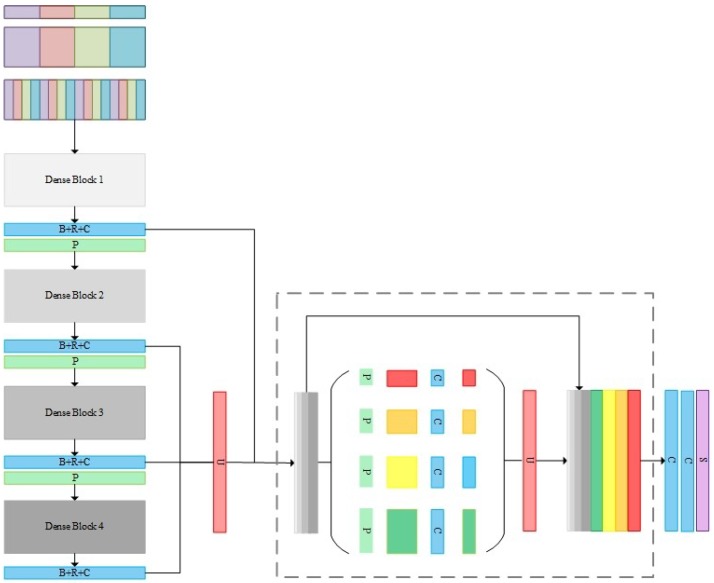
Architecture of the proposed dense pyramid network. B + R + C: Batch normalization + Rectified Linear Unit (ReLU) + Convolutional layer (kernel size: 1 × 1, stride: 1), where P: Pooling layer (kernel size: 2 × 2, stride: 2); U: Upsamling layer; C: Convolutional layer (kernel size: 3 × 3, stride: 1); S: Softmax layer.

The architecture of the proposed DPN included three main parts: (1) the group convolutions and the shuffling operation for multi-sensor data feature preservation; (2) the densely connected convolutions for high-level semantic feature extraction; (3) the pyramid pooling operation for multi-sensor and multi-resolution feature fusion. Furthermore, the median frequency balanced focal loss used in the training phase is presented. The architecture of the proposed DPN is shown in the Figure 2.

### 2.1. Group Convolutions and Channel Shuffling Operation

Multi-sensor data brought more information to the high-resolution aerial image classification task. For example, the digital surface model (DSM) contained the height information of Earth objects, which helped recognize buildings or trees. In the previous works, the popular way to use multi-sensor data was to stack them as one vector for the input of the network. Thus, the multi-sensor data fusion was accomplished at the first layer. However, the rough fusion operation by a convolutional layer may cause the information loss while brings classification ambiguity to certain objects. In this work, we proposed to perform group convolutions [21] on multi-sensor data to extract more features in channel-wise before feature fusion. Furthermore, in order to mitigate the reduction of information representation ability caused by the limited fraction of the input channels, channel shuffling operation [22] was followed to enhance the information flow between channels. The shuffling operation can be written as:

(1) $$shuffle(x)=flatten({x\prime }_{ng}^{T})$$

(2) $$x\prime =reshape{\left(x\right)}_{gn}$$

where *x* is the output of the group convolutions, *g* is the number of the groups of a convolution layer, and *n* is the filter number of each group. *Reshape* means reshaping the output vector in the group convolution layer into shape (*g*, *n*); *flatten* means flattening the output vector back as the input of dense blocks. In the proposed DPN, the group convolutional layer have four groups, of which each group has 16 filters.

### 2.2. Densely Connected Convolutions

Densely connected convolutions [23] are the high-level feature extractor of the DPN, of which each layer is connected to every other layer in a feed-forward fashion. The densely connected structure can resolve the vanishing gradient problem, take full advantage of all features, and strengthen feature propagation. Densely connected convolutions can be formulated as:

(3) $$y_{ijd\prime }^{l}=\sum_{d=1}^{D}\sum_{q=1}^{Q}\sum_{p=1}^{P}W_{pqd}x_{pqd}^{l}+b$$

(4) $$x^{l}=C([x^{1},x^{2},\ldots ,x^{l-1}])$$

Each filter has a series of learnable parameters which are arranged as a convolution kernel with size *P* × *Q* × *D*, where *P*, *Q*, and *D* represent length, width, and depth of the convolution kernel, respectively. The conversion of input image with a size of *N_i_* × *L_i_* × *D* to the convolutional layer output with a size of *N_o_* × *L_o_* × *D*, performed by a convolutional layer (a set of *D′* filters).

**Figure 3:**
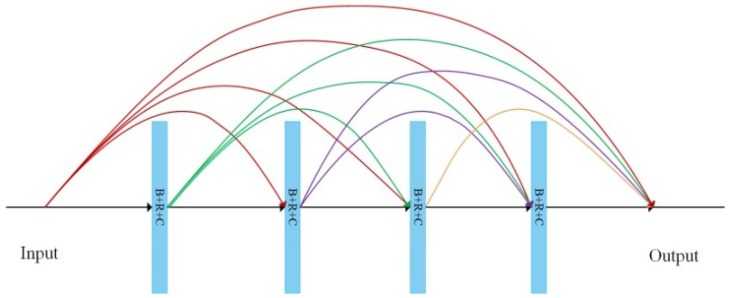
Architecture of a dense block with four layers. B + R + C: Batch normalization + ReLU + Convolutional layer (kernel size: 3 × 3, stride: 1).

The structure of an exemplary four-layer dense block is illustrated in Figure 3. Dense blocks are connected by a so-called bottleneck layer, which consists of one 1 × 1 convolutional layer (stride: 1) and one 2 × 2 average pooling layer (stride: 2), to reduce dimensions and integrate features of each layer. In this work, we apply four dense blocks to accomplish high level feature extraction, with each block containing 16 convolutional layers.

### 2.3. Pyramid Pooling Module

To provide high-level features with more semantic information, four deep dense blocks offered the network farther vision while suffering from a location information loss problem. Therefore, in our proposed DPN, we utilized not only a high-level feature but also lower-level feature to mitigate the recognition/localization tradeoff. The feature maps from the second, third, and last dense blocks were first up-sampled into the original image size and then fused through an effective global scenery prior manner, using a pyramid pooling module that combined two convolutional layers. Inspired by [19] and following our previous work [24], multi-resolution feature maps were concatenated at varying depths, since higher layer features of CNN actually gained more features. The pyramid pooling module [25] consisted of multi-scale pooling and up-sampling layers, as depicted in Figure 1’s dotted-line box. Different level features were then concatenated to create the final pyramid pooling global feature. In this case, the network obtained information with a different scale from various sub-regions of multi-resolution feature maps. We applied four scale pooling layer with kernel sizes {5 × 5, 10 × 10, 15 × 15, 30 × 30} for the Vaihingen dataset and 9 × 9, 18 × 18, 27 × 27, 54 × 54 for the Potsdam dataset, respectively. Additionally, two convolutional layers are followed to reduce dimensions and produce the final score maps of each class.

### 2.4. Median Frequency Balanced Focal Loss

**Figure 4:**
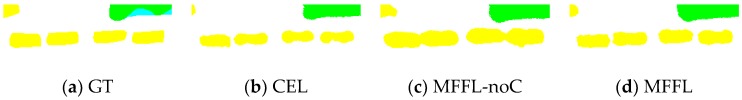
Results of an image patch with cars obtained from DPN, trained by different loss functions. Classes: Impervious Surface (white); low vegetation (cyan); tree (green); and car (yellow). GT: ground truth; CEL: result obtained from DPN trained by standard cross entropy loss; MFFL-noC: result obtained from DPN trained by median frequency balanced focal loss without constraint; MFFL: result obtained from DPN trained by median frequency balanced focal loss.

In order to down-weight the loss assigned to well classified samples and overcome the class imbalance problem of the high-resolution aerial dataset, we proposed to use median frequency balanced focal loss function to train our network. Focal loss [26] is based on cross entropy (CE) loss, which reshapes CE loss with a modulating factor that enforces the network to pay more attention to hard-classified samples. The focal loss is defined as follows:

(5) $$FL(p,q)=-\frac{1}{N}\sum_{n=1}^{N}\sum_{c=1}^{C}{\left(1-q_{c}^{\left(n\right)}\right)}^{\gamma }p_{c}^{\left(n\right)}\log(q_{c}^{\left(n\right)})$$

where $p_{c}^{\left(n\right)}$ is the one-hot label for class *c* of the *n*-th image patch in the current batch, $q_{c}^{\left(n\right)}$ is the output of softmax layer, *C* is the number of class, and *N* is the total number of training images in each batch. γ is a tunable parameter to make the network focus on hard-classified samples when it is greater than 0. In this work, we set the sample as 2, since it was found to bring the best performance. Furthermore, the focal loss is weighted by the pixel median frequency [27] with constraints of each class. The median frequency balanced focal loss can be written as:

(6) $$MFFL(p,q)=-\frac{1}{N}\sum_{n=1}^{N}\sum_{c=1}^{C}\omega_{c}{\left(1-q_{c}^{\left(n\right)}\right)}^{\gamma }p_{c}^{\left(n\right)}\log(q_{c}^{\left(n\right)})$$

where *ω_c_* is median frequency weight with constraint of the class *c*, and *f_c_* is the pixel frequency of the class c. The median frequency with a constraint is expressed as follow formula:

(7) $$\omega_{c}=\log\{[\frac{median(\left\{f_{c}|c\in C\right\})}{f_{c}}]+1\}$$

when we added the median frequency without constraints (marked as the contents in the medium brackets of Formula (7)) for each class directly, the overreacting problem for a certain class of small objects occurred. Figure 4 shows the results of an image patch with cars obtained from DPN, trained by different loss functions. Figure 4b is the result of the DPN trained by cross entropy loss, in which many car class pixels were mislabeled as roads and some car class pixels in shadow could not be detected. When we weighed the cross-entropy loss with the median frequency of each class (Figure 4c), most car pixels could be detected, but it came at the cost of many non-car class pixels to be mislabeled as cars (false positives). Therefore, in order to compromise the effect of the median frequency weights, we added the constraint to the median frequency and the results yield much better accuracy for cars, as shown in Figure 4d.

### 2.5. Training and Inference Strategy of DPN

Using an Adam optimizer, we trained the proposed DPN in order to optimize the median frequency and balance the focal loss [28,29,30]. We initialized parameters by normally distributing random variables. The initial learning rate was set to 10^−4^ and stepped down 10 times every 5 epochs. The batch size was 3. The training image patches of Vaihingen and Potsdam datasets were extracted with 50% overlap, and with sizes 256 × 256 pixels and 512 × 512 pixels, respectively. In order to mitigate the over-fitting problem caused by constraints of the training data, data augmentation was applied to the training set. The extracted image patches were first flipped vertically and horizontally, and then rotated 90°, 180°, and 270°.

In the testing phase, a sliding window was employed to the full tile testing images. Since the images in testing set were larger than 2000 × 2000 pixels, we needed to crop the testing images into smaller patches to fit the memory. A sliding window overlap was adopted to avoid the border inconsistent phenomenon. We set 75% of the overlapping size in the inference procedure, as the size proved to achieve better than in previous works [17,20].

## 3. Results

### 3.1. Dataset and Evaluation Metrics

We evaluated the proposed network, Vaihingen and Potsdam datasets provided by ISPRS [31], on the benchmark of high-resolution aerial image labeling. The Vaihingen dataset is comprised of 33 true orthophoto (TOP) (with an average size of 2494 × 2064) at a spatial resolution of 9 cm, of which 16 tiles are annotated with labels, and the other 17 tiles are reserved as hidden test set. All TOP tiles have near infrared (NIR), red (R), green (G) channels, together with digital surface models (DSMs) and normalized DSMs (nDSMs) [32]. The dataset was labeled into six classes, namely impervious surfaces, building, low vegetation, tree, car, and clutter. We selected 11 tiles (1, 3, 5, 7, 13, 17, 21, 23, 26, 32, 37) for training and 5 tiles (11, 15, 28, 30, 34) for validation. The other 17 hidden testing tiles (2, 4, 6, 8, 10, 12, 14, 16, 20, 22, 24, 27, 29, 31, 33, 35, 38) were additionally used for testing.

The other dataset we used was the Potsdam dataset. The Potsdam dataset consisted of 38 TOP tiles (of size 6000 × 6000) at a spatial resolution of 5 cm. Each tile had four bands, including NIR, R, G, blue (B) channels, and DSMs; nDSMs were also provided. Furthermore, 24 tiles were annotated with the same six classes as the Vaihingen dataset; the other 14 tiles were preserved as a hidden test set. We chose 18 tiles (2_10, 2_11, 3_10, 3_11, 4_10, 4_11, 5_10, 5_11, 6_7, 6_8, 6_9, 6_10, 6_11, 7_7, 7_8, 7_9, 7_10, 7_11) for training, and 6 tiles (2_12, 3_12, 4_12, 5_12, 6_12, 7_12) for validation. Furthermore, the other 14 tiles in the hidden testing set were selected for testing. 

(It is worth noting that the ground truth of the hidden test set was released in June 2018. The ISPRS 2D semantic labeling contest organizers will no longer update the evaluating results. Under these circumstances, we compute the same evaluation metrics of our results based on the released ground truth to compare our network with the other methods.)

In order to evaluate the overall performance and accuracy of each method, we computed the proportion of corrected labeled pixels. Moreover, the per-class F1 score is used to evaluate the performance for each class, which can be written as:

(8) $$F_{1}=2\times \frac{precision\cdot recall}{precision+recall}$$

where *precision* and *recall* can be calculated based on the confusion matrices. Precision is the true positive pixels divided by the sum of true positive and false positive pixels. Recall is the ratio of true positive pixels and the sum of true positive and false negative pixels. We also computed the average F1 score for all classes.

### 3.2. Ablation Study

In this section, we will first present whether the group convolutions and shuffling operation were found to achieve a better multi-sensor data fusion performance. Next, we will compare the proposed version with the DPN-noGS, which removed group convolutions and shuffling operations, and directly fed the input images (NIR-R-G-nDSMs) to dense blocks. Furthermore, we will assess the pyramid pooling module, which was replaced by a multi-layer perceptron [19] that compared the proposed version. Thus, we will check whether the pyramid pooling module can bring benefits to the multi-resolution feature fusion and improve the segmentation accuracy. At last, we will train DPN with the proposed median frequency balanced focal loss (DPN-MFFL), to study how the proposed loss affects the network performance.

Table 1 details the validated results of the approaches described above. We can observe that the proposed DPN architecture achieves higher accuracy than DPN without group convolutions and shuffling operation version, which indicates these two operations can help the network preserve more useful information from multi-sensor data than fuse them at the first layer directly. Meanwhile, DPN without the pyramid pooling module version was also outperformed by the proposed DPN. The MLP can learn to fuse multi-resolution feature maps in an effective way, but the pyramid pooling module shows greater capacity in feature fusion by different-region based context aggregation. Furthermore, when the DPN trained by using MFFL was compared with the proposed DPN trained with the standard cross entropy loss, most evaluation metrics increased, especially for class car, which indicated that the proposed MFFL can benefit the accuracy of small objects like class car.

**Figure 5:**
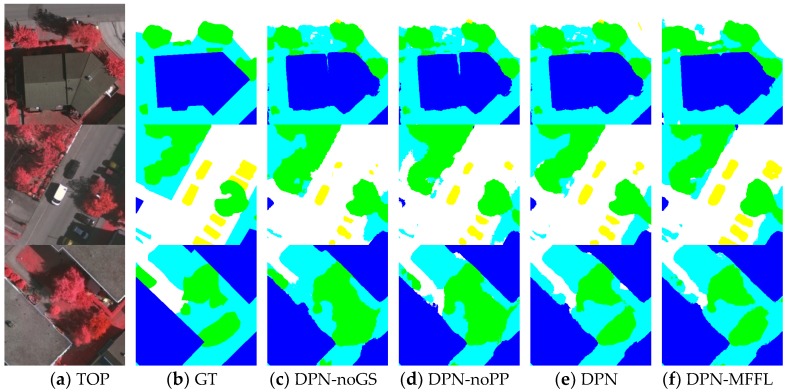
Semantic segmentation results for three image patches of Vaihingen validation set. The label includes six categories: impervious surface (imp surf, white); building (blue); low vegetation (low veg, cyan); tree (green); car (yellow); and clutter/background (red).

Figure 5 shows the visual comparisons on three image patches of the Vaihingen validation set. The first row of Figure 5 is an image patch of a building with a highly inconsistent roof. It also shows the results of the DPN without group convolutions and shuffling operation (GS), and a version without the PP module, demonstrating how they were effected by shadows on the roof. The GS preserved more information, while the PP module provided global contextual information. The proposed DPN labels the building more accurately than the original. In addition, the car class is difficult to label correctly because of its various colors and the influence of shadows. The second row of Figure 5 is an image patch with cars, and several of them are parked in the shadows of trees and buildings. The DPN-noGS and DPN-noPP fail to label the cars in shadows. The proposed DPN trained by standard cross entropy loss achieved a slight improvement in the car detection. Thus, the DPN trained by MFFL can label most cars in shadows more clearly and precisely. Trees and low vegetation in this dataset were prone to be confused by the networks, as shown in the third row of Figure 5, the DPN-noGS and DPN-noPP mislabel some low vegetation areas as trees whilst the proposed DPNs can provide a relatively proper segmentation results for these plants.

### 3.3. Comparison with Other Methods

#### 3.3.1. Vaihingen Dataset

In order to verify the performance, we evaluated the proposed DPN on the remaining 17 testing tiles of the Vaihingen dataset and compared it with methods on the benchmark test. The details of these methods are listed below:

(1) ‘DST_2’: The method (FCN + RF + CRFs + DSMs) is proposed in Ref. [14]. The CIR images and LiDAR data are processed and combined by a hybrid FCN, of which no down-sampling FCNs are employed to preserve resolution of the output. CRFs are utilized to further improve the segmentation accuracy as a post-processing step.
(2) ‘ONE_7’: The method (SegNet + DSMs/nDSMs) is proposed in Ref. [17]. Two SegNets are applied for both CIR images and synthetic data, which consist of DSMs, nDSMs, and the normalized difference vegetation index (NDVI) computed based on NIR and R channel.
(3) ‘INR’: The method (CNN + MLP + DSMs) is proposed in Ref. [19]. Authors derived a CNN framework to learn features and multi-resolution features which are inputted into MLP to learn how to fuse in an effective and flexible fashion. It is worth noting that DSMs are simply added as an extra band of input data.
(4) ‘CAS_Y3’: The method (PSPNet) is proposed in Ref. [25]. ResNet 101 is utilized as the feature extractor and pyramid pooling module is followed to learn global contextual information at multiple scales. No LiDAR data is used in this method, since the input of ResNet101 follows a 3-channel format.
(5) ‘BKHN-5’: The method (DenseNet + DSMs/nDSMs) is proposed in Ref. [23], where 67 layers of fully convolutional DenseNet is selected as a comparison method. This is because these 67 layers of fully convolutional DenseNet achieves the best performance when compared with 46 layers (‘BKHN-9’) and 103 layers (‘BKHN-8’); the 67 layers of fully convolutional DenseNet have a similar scale with the proposed DPN, which employs 64 layers of densely connected convolutions.
(6) DPN-MFFL: The method (DPN + Focal Loss + nDSMs) is proposed in this paper, where 64 layers of densely connected convolutions combined with a pyramid pooling module is employed to learn high-level features and accomplish the feature fusion through a global contextual prior manner. In addition, group convolutions and shuffling operation are adopted to preserve more information from multi-sensor data. In the training phase, the focal loss is optimized to mitigate the class imbalance problem.

The numerical results of these methods are exhibited in Table 2. As the table shows, the proposed DPN-MFFL outperforms the other methods in most per-class F1-scores, average F1-scores, and overall accuracy. In particular, the F1-score of class car achieves considerable improvements, due to the median frequency that balances focal loss in the training phase. 

“DST_2” and “ONE_7” are based on two classic models in semantic segmentation field, FCN and SegNet, respectively. These two methods propose to train the two networks on CIR images and LiDAR data separately, and then fuse features of the two models to predict the final score map of each class. Qualitatively, “ONE_7” achieves better performance than “DST_2”, which is owed to the multi-scale prediction and better fusion strategy of “ONE_7.” Furthermore, “ONE_7” is included in the Normalized Difference Vegetation Index (NDVI) feature. The NDVI is computed from NIR and R channels, which brings benefits to the vegetation class recognition. This explains the outstanding performance of class trees and low vegetation. However, this kind of strategy suffers from more than double the increase in trainable weights.

“INR” and “BKHN_5” add LiDAR data as an extra band of input, as well as fuse multi-sensor data at first layer. Network structures are therefore relatively simpler than the first two methods. However, fusing the multi-sensor data at the first layer may cause classification ambiguities in those classes without obvious height information. For example, the class car of “INR” and class low vegetation of “BKHN_5” show the lowest F1-score. “CAS_Y3” utilizes only CIR images to train the PSPNet (pre-trained ResNet101 as feature extractor), and the abandon of LiDAR data leads to its worse performance in class building and tree. The proposed DPN-MFFL outperforms the other methods by a considerable margin, especially when compared with “CAS_Y3” and “BKHN_5”, which have the same network components (pyramid pooling module and densely connected convolutions) as the proposed method. The DPN-MFFL exhibits the best performance in most evaluation metrics, which further indicates the rationality of our structural network design.

**Figure 6:**
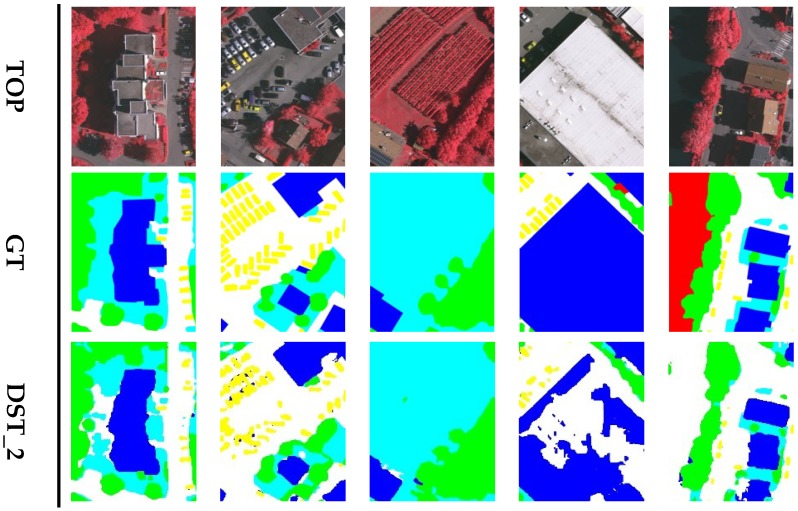

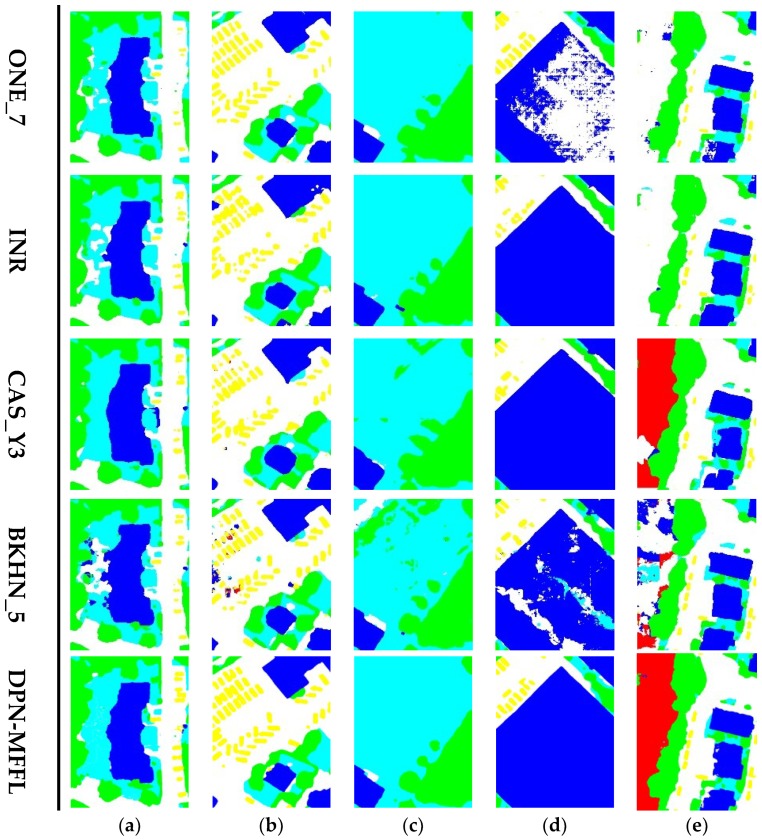
Qualitative comparison with other competitors’ methods on ISPRS Vaihingen challenge online test set. The label includes six categories: impervious surface (white)l building (blue); low vegetation (cyan); tree (green); car (yellow); and clutter (red). Five image patches included: (**a**) low vegetation in the shadow of building; (**b**) cars in a parking lot; (**c**) trees and low vegetation; (**d**) a large building; and (**e**) city waters.

As can be seen from the Figure 6a, shadows pose challenges for semantic segmentation. Most comparison methods mislabeled the low vegetation under the shadows in different degrees. Global contextual information was provided using the pyramid pooling module, “CAS_Y3”, and the proposed “DPN-MFFL” achieved more accurate segmentation results after shadow interference.

The class imbalance problem usually leads to low segmentation accuracy of class car, since cars are relatively small when compared with other earth objects and they account for fewer pixel numbers in the training set. We can observe in Figure 6b that most comparison methods performed poorly in this class. With the help of the median frequency balanced focal loss, the proposed DPN-MFFL handles the class imbalance problem properly and labels cars accurately.

In this dataset, trees and low vegetation, along with cement-roofed buildings and roads, had similar colors. Therefore, networks confuse and mislabel these classes (see Figure 6c,d). The proposed DPN-MFFL can better distinguish these confusing earth objects. The large intra-class differences make it difficult to label class clutter correctly (see Figure 6e). The proposed DPN-MFFL can label the city water of class clutter accurately and completely, while other methods mislabeled this kind of clutter as impervious surface or buildings. Thus, the proposed method can provide fine-grained segmentation maps with better visual quality.

#### 3.3.2. Potsdam Dataset

We also evaluated our method on the Potsdam testing dataset. Because the methods on the Potsdam benchmark test were not the same as the Vaihingen benchmark test, we chose methods similar to the methods selected in Vaihingen benchmark test to compare with the proposed DPN-MFFL. The detail of these comparison methods are listed below:

(1) ‘UZ_1’: The method (CNN + Deconvolution + nDSMs) is proposed in Ref. [11]. The architecture followed up-sample-then-down-sample paradigm. It learns the high-level features by convolutions, and then learns to up-sample the down-sampled features back to original size by means of deconvolutions. The nDSMs are treated as an extra band of the input data.
(2) ‘RIT_L7’: The method (FCN + CRFs + nDSMs) is proposed in Ref. [12]. The authors train a FCN-8s on CIR images and a logistic regression by hand-craft features derived from nDSMs and CIR images. The results of two architectures are then fused by using a higher order CRFs to obtain the final probabilistic graphs.
(3) ‘RIT_2’: The method (SegNet + nDSMs) is proposed in Ref. [33]. Two SegNets are trained on CIR images and synthetic data separately, and the features are then fused at the early stage to reduce the memory cost.
(4) ‘DST_5’: The method (FCN + RF + CRFs + DSMs) is proposed in Ref. [14]. Same as ‘DST_2’ described in Section 3.3.1.
(5) ‘CAS_Y3’: The method (PSPNet) is proposed in Ref. [25]. Same as ‘CAS_Y3’ described in Section 3.3.1.

Table 3 details the numerical results of each method. In terms of overall accuracy and the average F1-score, the DPN-MFFL achieves the best results when compared with the other methods in the leaderboard. As for the per-class F1-scores, the impervious surface of the DPN-MFFL is slightly surpassed by ‘DST_5’. But for most classes, F1-scores of the proposed method are improved when compared with the other methods.

**Figure 7:**
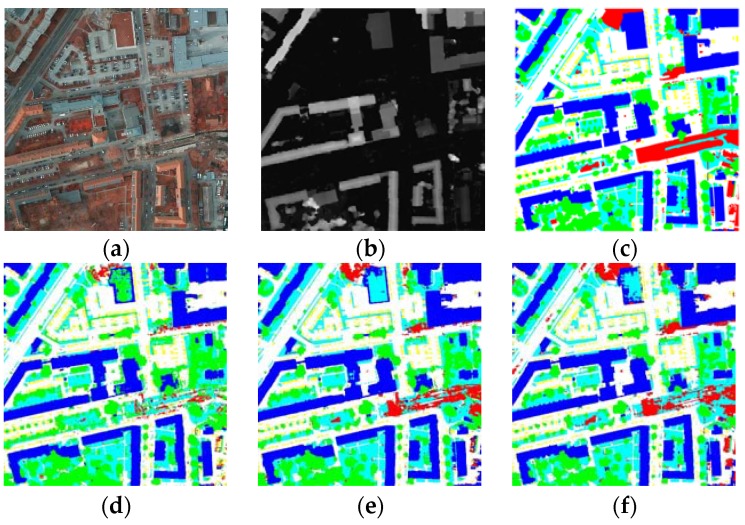

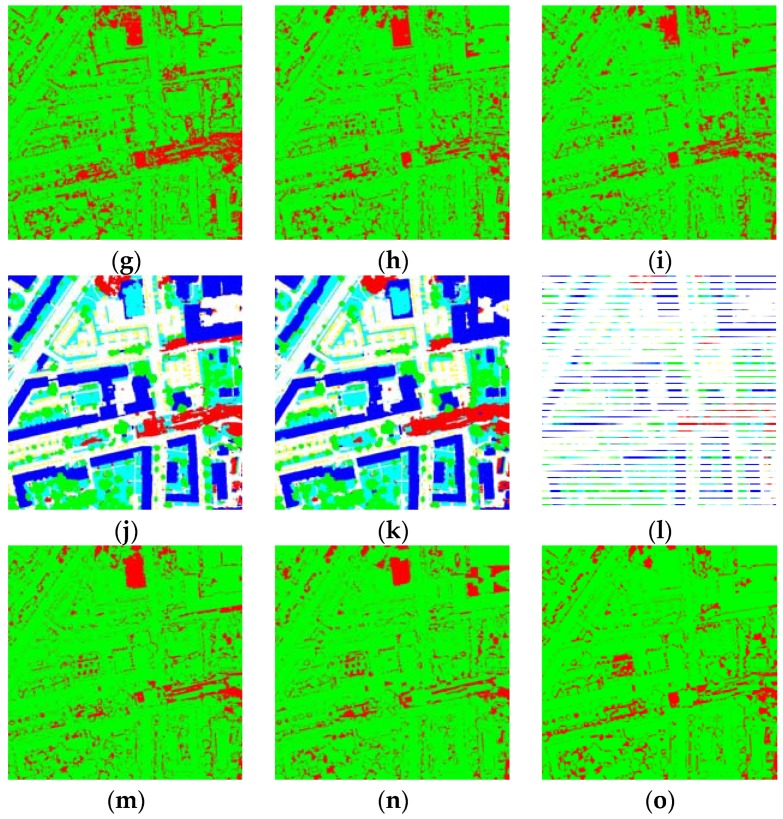
Qualitative comparison with other competitors’ methods on ISPRS Potsdam challenge online test set. The label includes six categories: impervious surface (white), building (blue), low vegetation (cyan), tree (green), car (yellow) and clutter/background (red). (**a**) TOP tile; (**b**) nDSM; (**c**) ground truth; (**d**–**f**) prediction results of ‘UZ_1’, ‘RIT_L7’ and ‘RIT_2’; (**g**–**i**) red-green maps of ‘UZ_1’, ‘RIT_L7’ and ‘RIT_2’; (**j**–**l**) prediction results of ‘DST_5’, ‘CAS_Y3’ and the proposed DPN-MFFL; and (**m**–**o**) red-green maps of ‘DST_5’, ‘CAS_Y3’ and the proposed DPN-MFFL.

Figure 7 exhibits the full tile prediction comparisons. The red-green maps (green: correctly labeled pixels, red: mislabeled pixels) clearly display the performance differences among each method. As can be seen from the pictures, the proposed DPN-MFFL provides a more accurate classification map. In particular, when dealing with some hard-classified pixels like buildings with roof lawn and clutters in complex pattern, the proposed method shows better performance.

## 4. Conclusions

In this paper, we proposed a new DPN to perform semantic segmentation of high-resolution aerial images. The adopted architecture consists of three major parts: group convolutions and shuffling operation for multi-sensor feature preservation; densely connected convolutions for high-level feature extraction; pyramid pooling module combined with two convolutional layers for multi-resolution (high-level and low-level) feature fusion. In particular, median frequency balanced focal loss is utilized to train the network to mitigate the class imbalance problem. Experimental results in the Vainhingen and Potsdam datasets illustrate the advantages of the proposed framework. Without any post-processing steps, the proposed DPN achieved better performance when compared with some state-of-the-art methods.

## Figures and Tables

**Table 1:** Experimental results on the Vaihingen validation dataset. DPN-noGS: DPN without group convolutions and shuffling operation version; DPN-noPP: DPN without the pyramid pooling module version; DPN: the proposed DPN trained by standard cross entropy loss function; DPN-MFFL: DPN trained by median frequency balanced focal loss.

Methods	Imp. Surf.	Build	Low Veg.	Tree	Car	Aver. F1	OA
DPN-noGS	90.95	94.68	79.29	88.46	82.32	87.14	88.74
DPN-noPP	90.08	94.95	79.01	88.16	83.33	87.11	88.35
DPN	91.33	**95.53**	**80.50**	88.49	83.90	87.95	89.19
DPN-MFFL	**91.49**	95.43	80.47	**88.72**	**85.69**	**88.36**	**89.26**

The bold values represent the best value.

**Table 2:** Experimental results on the (ISPRS) Vaihingen challenge online test set.

Methods	Imp. Surf.	Build	Low Veg.	Tree	Car	Aver. F1	OA
DST_2 [14]	90.5	93.7	83.4	89.2	72.6	85.9	89.1
ONE_7 [17]	91.0	94.5	**84.4**	**89.9**	77.8	87.5	89.8
INR [19]	91.1	94.7	83.4	89.3	71.2	85.9	89.5
CAS_Y3 [25]	91.0	93.3	82.2	88.2	71.3	85.2	88.7
BKHN_5 [23]	91.4	94.3	81.9	88.5	78.4	86.9	89.1
DPN+MFFL	**91.9**	**95.1**	84.0	89.0	**81.8**	**88.4**	**90.0**

The bold values represent the best value.

**Table 3:** Experimental results on the ISPRS Potsdam challenge online test set.

Methods	Imp. Surf.	Build	Low Veg.	Tree	Car	Aver. F1	OA
UZ_1 [11]	89.3	95.4	81.8	80.5	86.5	86.7	85.8
RIT_L7 [12]	91.2	94.6	85.1	85.1	92.8	89.8	88.4
RIT_2 [33]	92.0	96.3	85.5	86.5	94.5	91.0	89.4
DST_5 [14]	**92.5**	**96.4**	86.7	**88.0**	94.7	91.7	90.3
CAS_Y3 [25]	92.2	95.7	87.2	87.6	95.6	91.7	90.1
DPN-MFFL	92.4	**96.4**	**87.8**	**88.0**	**95.7**	**92.1**	**90.4**

The bold values represent the best value.
